# Spatial distribution of heavy metals in soil, water, and vegetables of farms in Sanandaj, Kurdistan, Iran

**DOI:** 10.1186/s40201-014-0136-0

**Published:** 2014-11-14

**Authors:** Afshin Maleki, Hassan Amini, Shahrokh Nazmara, Shiva Zandi, Amir Hossein Mahvi

**Affiliations:** Kurdistan Environmental Health Research Center, Kurdistan University of Medical Sciences, Sanandaj, Iran; Department of Epidemiology and Public Health, Swiss Tropical and Public Health Institute (Swiss TPH), Socinstrasse 57, 4002 Basel, Switzerland; University of Basel, Petersplatz 1, 4003 Basel, Switzerland; Department of Environmental Health Engineering, School of Public Health, Tehran University of Medical Sciences, Tehran, Iran; Center for Solid Waste Research (CSWR), Institute for Environmental Research (IER), Tehran University of Medical Sciences, Tehran, Iran

**Keywords:** Coriander, Dill, GIS, Heavy metals, Iran, Mapping, Radish, Soil, Vegetables, Water

## Abstract

**Background:**

Heavy metals are ubiquitous elsewhere in nature and their measurement in environment is necessary to develop health management strategies. In this study, we aimed to find out concentrations and spatial patterns of heavy metals in main farms of Sanandaj in Kurdistan, Iran.

**Methods:**

Over May to October 2012, six farms were selected to analyze concentrations and spatial patterns of several heavy metals, namely aluminum (Al), arsenic (As), cadmium (Cd), cobalt (Co), chromium (Cr), copper (Cu), nickel (Ni), lead (Pb), and zinc (Zn) in their soil, irrigation water, and edible vegetables. Overall, 36 samples of soil and water and 72 samples of vegetables including coriander (*Coriandrum sativum*), dill (*Anethum graveolens*), radish (*Raphanus sativus*) root and radish leaf were collected. The concentrations of metals were determined by inductively coupled plasma optical emission spectrometry. The spatial surfaces of heavy metals were created using geospatial information system.

**Results:**

The order of metals in soil was Al > Zn > Ni > Cu > Cr > Pb > Co > As > Cd while in water it was Cr > Co > Zn > Pb > Cu > Ni > Al = As = Cd. The order of heavy metals in vegetables was Al > Zn > Cu > Cr > Ni > Pb > Co > As > Cd. Totally, the minimum concentrations of Al, Cu, Pb, and Zn were found in radish root while the maximum of Al, Co, Cr, and Ni were found in radish leaf. The minimum concentrations of Cd and Cr and maximum concentrations of Cu and Zn were also deciphered in dill. Noteworthy, coriander had the minimum concentrations of Co and Ni. The concentrations of Cr and Pb in vegetables were more than maximum allowable limits of the Food and Agriculture Organization (FAO) and the World Health Organization (WHO).

**Conclusion:**

In summary, albeit the concentrations of heavy metals in soil and water samples were below FAO and the WHO standards, vegetables were contaminated by chromium and lead.

## Background

Heavy metals—those with specific density of about 4-5 g/cm^3^ or higher—are actually ubiquitous elsewhere in nature. In fact, there are several sources of contamination, namely natural, agricultural, domestic, atmospheric, and other sources [1]. However, tremendous application and utilization of heavy metals in more recent years through industries caused steep emissions to our environment [2]. The main pathways of humans’ exposure to these elements are through air, water, soil and hence agricultural crops [3].

The health effects of heavy metals have been known since ancient history [2]. In essence, toxicity and/or carcinogenicity of heavy metals, such as lead, arsenic, mercury, cadmium, and nickel have been reported in numerous studies [4-10], even in low dose exposures [11]. In brief, they may cause kidney, lung, nervous system, and skeletal damages, such as itai-itai disease [12-15]. Diminished intellectual capacity, gastrointestinal symptoms, coronary heart disease, variety of cancers, such as renal, skin, and bladder cancers, and even death have been also associated with chronic and/or acute exposures to heavy metals [3,16-18].

Some of heavy metals, such as iron, copper, and zinc are present in trace amounts (10 mg/kg, or mg/l) or ultra-trace amounts (1 μg/kg, or μg/l) in environmental matrices as they are essential for biochemical and/or physiological functions. However, quantities beyond the requirement of living organism may lead to its toxicity [1].

Overall, majority of heavy metals daily intake in humans is from diet, mainly through crop plants [8,19]. It is well recognized now that certain species of plants tend to bioaccumulate heavy metals [20-23]. For instance, Maleki and Alasvand Zarasvand [24] reported that lead, copper, chromium, and cadmium were present in edible vegetables, such as leek, sweet basil, parsley, garden cress, and tarragon in Sanandaj, Iran beyond the allowable national standards. In addition, Mohajer et al. [25] declared that most plants grown in Isfahan, Iran had heavy metals more than international standards in which 48 and 75 percent of vegetable samples had concentrations of cadmium and lead beyond the Food and Agriculture Organization (FAO) and the World Health Organization (WHO) guidelines. Eslami et al. [26] determined high levels of cadmium and lead in parsley, leek, radish leaves and roots, and sweet basil, which were well above the maximum threshold values recommended by FAO and the WHO. Pourang and Noori [27] also analyzed heavy metals in various plant species of an agricultural area near Tehran and reported that chromium, cadmium, and lead were substantially above national and international guidelines in most of vegetables, especially in onions. Cadmium and lead were again principal culprits in cucumbers and bell peppers grown in greenhouses of Qom, Iran [28]. Besides, there are many similar studies from China, Ghana, and other countries that measured heavy metals in agricultural soils, irrigation water and/or their crops [29-33].

The excess presence of heavy metals in edible vegetables that people consume, as reported by numerous researchers, may pose a health threat to general population. Therefore, it is essential to monitor their concentrations in vegetables. Moreover, measurement of heavy metals in irrigation water and also agricultural soil may help to better understand sources of contamination and consequently develop effective management strategies. The application of Geospatial Information System (GIS) technology also has been proposed by other researchers to identify spatial distribution of trace elements and sources of pollution [34,35]. Therefore, in this study, the authors aimed to decipher levels and spatial patterns of heavy metals in soil, irrigation water, and edible vegetables of farms in Sanandaj, Kurdistan, Iran.

## Materials and methods

### Study area and sampling sites

**Figure 1:**
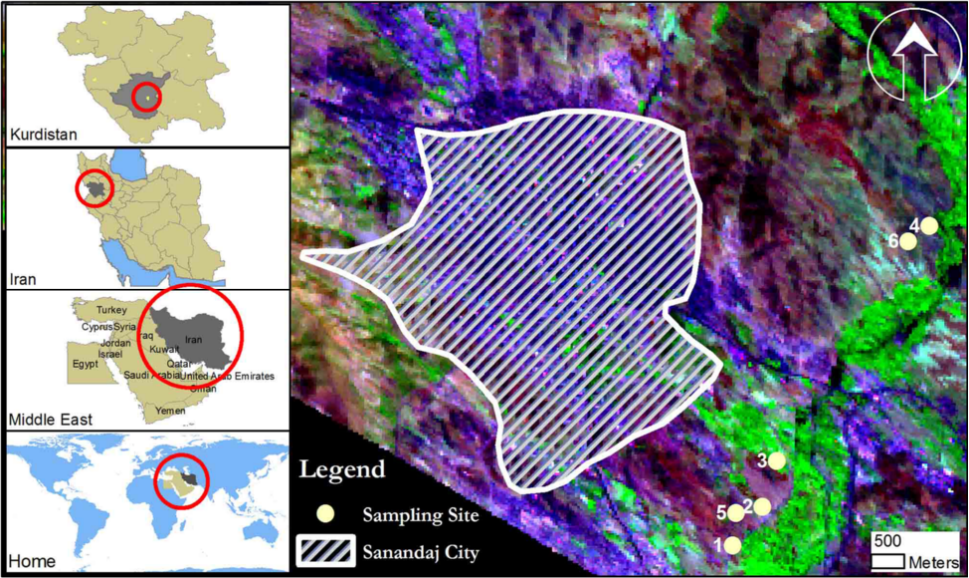
**Study area and location of sampling sites in Sanandaj, Kurdistan, Iran.**

Sanandaj is capital of Kurdistan province in North West of Iran. The city is developing and non-industrialized [36]. However, there are various small industries and workshops, such as welding of metals and plating, small tanning industries, etc., which are mainly located in southeast region. There are several farmland areas in outskirts of the city near Sanandaj’s small river, located also in southeast region (Figure 1). Nevertheless, there are six major farms for cultivating vegetables around the city and we included all of them in the study. Over May to October 2012, we selected these six farms to analyze levels and spatial distribution of several heavy metals, namely aluminum (Al), arsenic (As), cadmium (Cd), cobalt (Co), chromium (Cr), copper (Cu), nickel (Ni), lead (Pb), and zinc (Zn) in soil, irrigation water, and edible vegetables that they produce. The sampling was done over this period as it is suitable for planting and harvesting of vegetables in Sanandaj.

### Soil sampling

Overall, 18 samples of soil were collected bimonthly during study period (6 farms × 3 times). For each sample at each farm, a mixture of sub-samples was collected to form a composite soil sample to be representative of the whole farm. In detail, a hole of 25 cm was dug using a clean stainless steel trowel and three to four slices of soil from top to bottom were sampled and preserved in a clean bag. The bag was labeled and transferred to the laboratory [37,38].

### Irrigation water sampling

The farms were irrigated by groundwater. Overall, 18 samples of water were collected bimonthly during study period. In detail, sampling was done using a 500 ml washed polypropylene bottle. Noteworthy, 1 ml of nitric acid was added to the samples to avoid microbial activity [38,39].

### Vegetable sampling

The four commonly produced vegetables of these farms were selected for sampling including coriander (*Coriandrum sativum*), dill (*Anethum graveolens*), radish (*Raphanus sativus*) root and radish leaf. Overall, 72 samples of vegetable were compiled bimonthly during study period (6 farms × 4 vegetables × 3 times). A mixture of six sub-samples at various distances (10 m, 30 m, 50 m, 70 m, 120 m and 140 m) was collected for each sample to form a composite sample of vegetables at each farm [40].

### Analytical methods

The soil samples were oven dried at 80°C at laboratory until stable weights were obtained. Thereafter, they were crushed using a porcelain mortar with a pestle and mixed thoroughly until they could be sifted through a 10 mesh sieve. For heavy metal analyses, about 100 g of each soil sample was separated out, transferred to a beaker, and as stated in the SW-846, 3050 method of EPA, analytical degree of HNO_3_ and H_2_O_2_ (30%) were applied for soil digestion.

The vegetable samples were transferred to laboratory and washed with distilled water to remove mulch or soil. In order to remove excess moisture, samples were air dried for approximately two weeks. Afterwards, using a porcelain mortar and a pestle, samples were grounded until they could be sifted through a 10 mesh sieve. For heavy metal analyses, about 1 g of each sample was separated out and transferred to a Pyrex beaker. Then, 10 ml of mixture of acids including HNO_3_, HClO_4_ and H_2_SO_4_ in ratio of 1:1:1 was added to the beaker and maintained at laboratory for 24 hours. After this lag, the beaker was heated at 95°C on hot plate until volume was reduced to 10 ml. Again, 10 ml of acid mixture was added and until reaching to 4 ml was heated. Afterwards, 50 ml of deionized water was added and the digest was filtered. Finally, 100 ml of solution was made by adding double deionized water [38,40].

The concentration of heavy metals in soil, irrigation water, and vegetables were determined by inductively coupled plasma optical emission spectrometry (ICP-OES) (Model SPECTRO ARCOS, SPECTRO Inc.; Germany). In detail, torch type of apparatus was flared end EOP Torch 2.5 mm; detector was CCD; nebulizer type was cross flow; nebulizer flow was 0.85 l/min; plasma power was 1400 W; coolant flow was 14.5 l/min; and pump rate was 30 RPM. Meanwhile, physicochemical characteristics of soil including pH, EC, total neutralizing value, organic carbon, K, P, soil texture, bulk density, and soil particle percent were analyzed according to the standard methods.

### The GIS analysis

All geographic coordinates of sampling points were recorded using a Global Positioning System (GPS) device (Garmin, Garmin International Inc.; Kansas, USA). Next, a GIS was established and using Spatial Analyst extension to ESRI’s ArcMap 9.3 GIS (ESRI, Redlands, CA, USA), spatial surfaces of heavy metals in soil and vegetables were created by interpolation method. The applied interpolation method was Inverse Distance Weighted (IDW).

### Statistical analysis

Descriptive statistics, such as minimum, 25%ile, median, 75%ile, and maximum were presented by box plots. In addition, mean and standard deviation were calculated. We calculated all statistical analyses by STATA software (STATA Corp., TX, USA).

## Results

### Soil heavy metals

The concentrations of heavy metals in soil samples including Al, As, Cd, Co, Cr, Cu, Ni, Pb, and Zn are shown in Table 1.

**Table 1:** **Descriptive statistics of heavy metals in Sanandaj’s soil samples and the international maximum allowable standards**

**Heavy metal (mg/kg)**	**Al**	**As**	**Cd**	**Co**	**Cr**	**Cu**	**Ni**	**Pb**	**Zn**
Mean	4139.5	3.8	0.10	14.8	28.7	42.6	47.9	25.4	77.2
Median	4126.5	3.8	0.10	14.3	29.1	38.7	48.1	26.6	77.8
Standard deviation	84.9	0.6	0.06	1.5	1.9	14.9	2.7	3.1	21.6
Min	4045.0	3.0	0.00	13.4	25.7	27.9	43.3	21.3	52.9
Max	4278.0	4.6	0.20	17.4	30.6	61.1	51.7	28.2	100.6
Austria standard [41]	-	50	5	50	100	100	100	100	300
The European Union standard [42]	-	-	3	-	150	140	75	300	300

**Figure 2:**
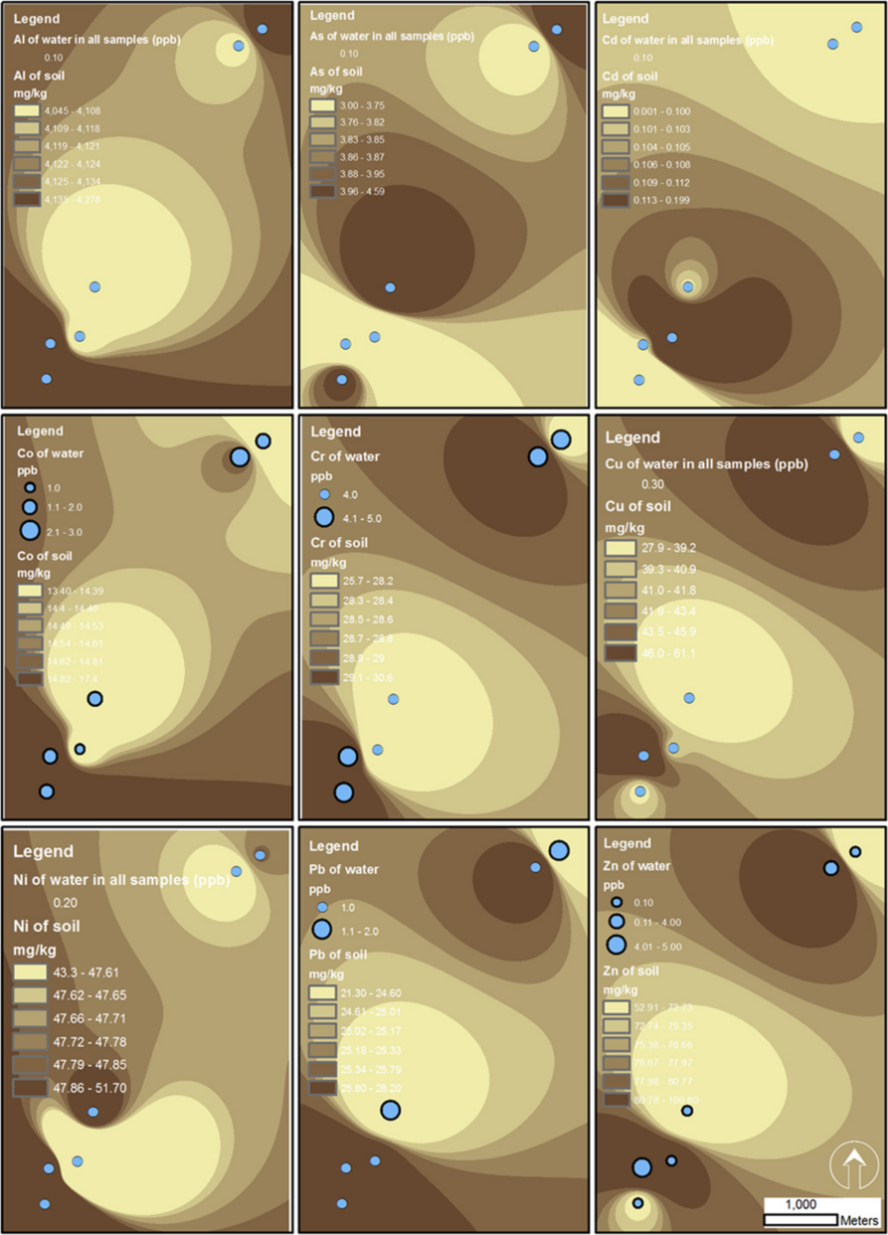
**Spatial patterns of heavy metals in soil and irrigation water of farms in Sanandaj, Kurdistan, Iran.**

The order of heavy metals presence in soil samples was as follow: Al > Zn > Ni > Cu > Cr > Pb > Co > As > Cd. Interestingly, heavy metals of soil in none of the cases exceeded international standard levels, such as Austria, and the European Union standards (Table 1). Results of physicochemical characteristics of soil samples are shown in Table 2. Importantly, pH of soil samples in all cases was circum-neutral to alkaline with the minimum of 7.8 and the maximum of 8.1 (Table 2). Figure 2, meanwhile, shows spatial pattern of measured heavy metals in soil samples of farms in Sanandaj, Kurdistan, Iran.

**Table 2:** **Physicochemical characteristics of soil samples in farms of Sanandaj, Kurdistan, Iran**

**Site**	**pH**	**EC (dS/m)**	**Total neutralizing value (%)**	**Organic carbon (%)**	**K (ppm)**	**P (ppm)**	**Soil texture**	**Bulk density (g/cm** ^**3**^ **)**	**Soil particle percent (%)**		
									**Clay**	**Silt**	**Sand**
1	8.1	0.622	9.75	1.54	261	11.7	Silty clay	-	47.5	50.7	1.8
2	7.9	0.954	13.5	1.9	323	29.2	Silty clay	1.25	42.5	43.2	14.3
3	7.9	0.726	12.25	0.2	527	25.2	Silty clay	1.21	50	41.6	8.4
4	7.9	0.711	11.5	1.3	512	22.7	Silty clay	1.21	49.2	43.7	7.1
5	7.8	0.610	24.25	1.47	302	10.14	Clay	1.26	47.5	23.2	29.3
6	7.8	0.625	25.45	1.2	337	12.2	Clay	1.25	48	24.5	27.5

### Irrigation water heavy metals

Table 3 shows the concentrations of heavy metals in water samples of farms in Sanandaj, Kurdistan, Iran. The order of heavy metals presence in water samples was as follow: Cr > Co > Zn > Pb > Cu > Ni > Al = As = Cd. The concentrations of Al, As, Cd, Cu, and Ni were similar for the six farms. As shown in Table 3, heavy metals of water samples in none of the cases exceeded international standard levels, such as Austria standard. Additionally, results of physicochemical characteristics are shown in Table 4. The pH of water samples in all cases was also circum-neutral to alkaline with the minimum of 7.6 and the maximum of 8.1 (Table 4). Meanwhile, spatial patterns of measured heavy metals in water samples of farms are shown in Figure 2 by graduate symbols.

**Table 3:** **Descriptive statistics of heavy metals in Sanandaj’s irrigation water samples and the international maximum allowable standards**

**Heavy metal (ppb)**	**Al**	**As**	**Cd**	**Co**	**Cr**	**Cu**	**Ni**	**Pb**	**Zn**
Mean	0.1	0.1	0.1	2.0	4.7	0.3	0.2	1.3	1.6
Median	0.1	0.1	0.1	2.0	5.0	0.3	0.2	1.0	0.1
Standard deviation	0.0	0.0	0.0	0.6	0.5	0.0	0.0	0.5	2.3
Min	0.1	0.1	0.1	1.0	4.0	0.3	0.2	1.0	0.1
Max	0.1	0.1	0.1	3.0	5.0	0.3	0.2	2.0	5.0
Austria standard [41]	5000	100	10	50	100	200	200	5000	2000

**Table 4:** **Physicochemical characteristics of irrigation water samples in farms of Sanandaj, Kurdistan, Iran** ^**a**^

**Site**	**pH**	**EC (μS/cm)**	**TDS (mg/l)**	**Total hardness (mg/l CaCO**_**3**_ **)**	**Turbidity (NTU)**	**Total alkalinity (mg/l CaCO**_**3**_ **)**
1	8.1	585	379	280	5.0	126
2	7.9	479	312	208	3.1	130
3	7.6	610	396	312	2.5	140
4	7.6	600	388	300	2.4	137
5	7.6	732	477	440	1.2	166
6	7.7	715	465	432	1.0	170

^a^Reprint from Maleki et al. [43] with written permission. *Abbreviations:* *EC* Electrical Conductivity, *TDS* Total Dissolved Solids, *NTU* Nephelometric Turbidity Units.

### Vegetables heavy metals

**Figure 3:**
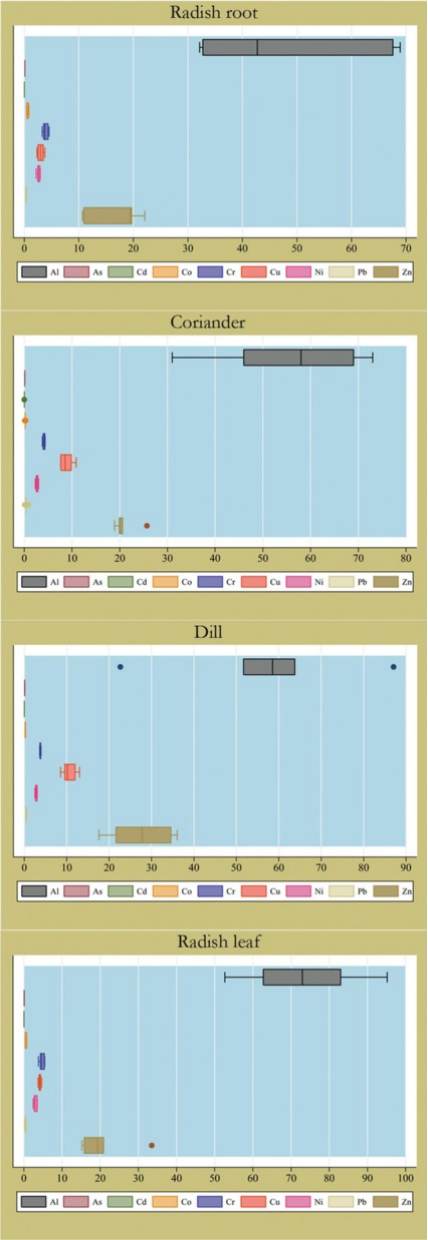
**Heavy metals of vegetables in farms of Sanandaj, Kurdistan, Iran.**

**Figure 4:**
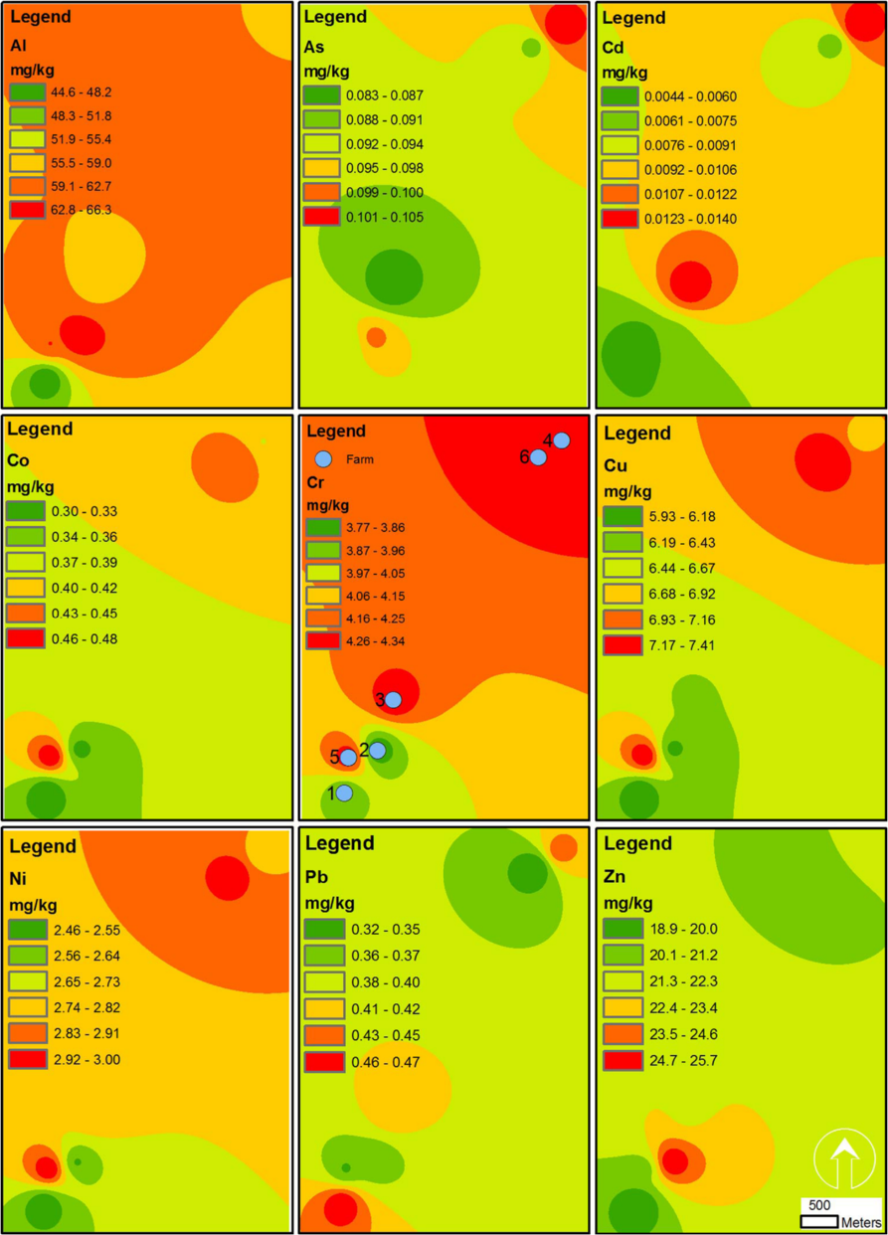
**Spatial patterns of heavy metals in vegetables of farms in Sanandaj, Kurdistan, Iran.**

Table 5 tabulated the mean concentrations of heavy metals in vegetable samples of farms in Sanandaj, Kurdistan, Iran. Besides, descriptive statistics including the minimum, 25%ile, median, 75%ile, and maximum of heavy metals in vegetables are illustrated in Figure 3 as box plots. The order of heavy metals presence in coriander and dill samples were similar and as follow: Al > Zn > Cu > Cr > Ni > Pb > Co > As > Cd. The order in radish leaf was as Al > Zn > Cr > Cu > Ni > Pb > Co > As > Cd and in radish root samples was as Al > Zn > Cr > Cu > Ni > Co > Pb > As > Cd. Overall, the order of heavy metals for vegetables was as follow: Al > Zn > Cu > Cr > Ni > Pb > Co > As > Cd. In general, the minimum concentrations of Al, Cu, Pb, and Zn were found in radish root while the maximum of Al, Co, Cr, and Ni were found in radish leaf. The minimum concentrations of Cd and Cr and the maximum concentrations of Cu and Zn also were deciphered in dill. Noteworthy, coriander had the minimum concentrations of Co and Ni (Table 5). Figure 4 shows spatial distribution of measured heavy metals in vegetable samples of farms in Sanandaj, Kurdistan, Iran.

**Table 5:** **Mean concentrations of heavy metals in Sanandaj’s vegetable samples and maximum allowable standard** ^**a**^

**Vegetable**	**Mean heavy metal (mg/kg)**								
	**Al**	**As**	**Cd**	**Co**	**Cr**	**Cu**	**Ni**	**Pb**	**Zn**
Coriander	55.8	0.10	0.010	0.2	4.2	8.9	2.6	0.4	20.9
Dill	57.0	0.10	0.005	0.2	3.8	10.6	2.8	0.4	27.6
Radish leaf	73.3	0.10	0.010	0.5	4.7	4.2	3.0	0.4	20.7
Radish root	47.8	0.10	0.010	0.6	3.9	3.0	2.6	0.3	17.0
Total	58.5	0.10	0.010	0.4	4.2	6.7	2.8	0.4	21.5
FAO and the WHO standard [44]	-	0.43	0.200	50	2.3	73.5	67.9	0.3	99.4

^a^Partly reprint from Maleki et al. [43] with written permission.

## Discussion

In this research, we characterized the concentrations of several important heavy metals in agricultural soil, irrigation water, and vegetable crops of six farms in Sanandaj, Kurdistan, Iran. We further depicted spatial distribution of these heavy elements in the studied area.

In general, we know now from our wide literature that levels of each heavy metal in soil is controlled by several interactive complex factors, such as pH, organic matter, cation exchange capacity, clay constitute of soil, and concentrations of aluminum and iron content [45]. More importantly, these factors, especially pH, would affect bioaccessibility and bioaccumulation of these heavy elements in plants. While aluminum is one of the most abundant elements in soil, its availability for crops is dependent on pH [46,47]. It is believed that soluble aluminum, which is absorbable by plants, is present in soil pH below 5.0, where rarely this pH is suitable for agriculture [48]. Noteworthy, aluminum has been classified as a *contaminant of potential concern* only in soils with a pH of 5.5 or less [46]. This may be the reason why no standard have been set for aluminum in soil (Table 1). However, the unexpected result was that aluminum became the first rank in vegetables of Sanandaj (Table 5 and Figure 3) while pH of soil was circum-neutral to alkaline (Table 2), which means aluminum has been absorbable in soil pH above 5.5. Meanwhile, the minimum and the maximum concentrations of aluminum among the four tested vegetables, respectively, were found in radish root and radish leaf, which means radish leaf may contribute to the maximum intake of aluminum to Sanandaj’s residents through consumption of tested vegetables. Interestingly, the concentration of aluminum in irrigation water samples, which was 0.1 ppm, is much lower than Austria's standard for irrigation water (5000 ppm).

The concentrations of all measured heavy metals in soil and irrigation water were below maximum allowable international standards, while in vegetables the concentrations of chromium (4.2 mg/kg) and lead (0.4 mg/kg) were more than the allowable limits, which are 2.3 and 0.3 mg/kg, respectively (Table 5). The presence of small industries/workshops in the proximity of the farms may be sources of these contaminations. However, this interpretation will be dubious as the concentrations of all heavy metals in soil and water were below the maximum allowable standards. Another reason for lead contamination of vegetables may be that these farms are located near major roads, which may cause air pollution and produce fine particles containing lead. However, it is more than 10 years that pb has been removed from petrol of Iran because of health-related issues [49]. The other interpretation, which is more credible, is presence of Middle Eastern Dust (MED) events in this region [36,50]. In fact, during last decade the welkin of about 11 provinces of Western and South Western Iran have dramatically changed because of MEDs, which is believed originate from deserts of Iraq and Syria [50,51]. Although we could not find any study to date in Sanandaj that evaluate ionic composition of the MEDs, especially heavy metal composition, and as the samples of soil and water were not contaminated by heavy metals, the contamination of vegetables by chromium and lead may be due to the MEDs. In any case, chromium is toxic for most plants and affect seed germination [52,53]. Moreover, chromium compounds have been considered as carcinogen for humans [54]. Long-term exposure to lead, also, is associated with decreases of intelligence capacity in children [49]. Thus, it is essential to inform Sanandaj's population that chromium and lead contaminated vegetables of these farms. In detail, none of the sampled vegetables was safe to human consumption. However, the order of presence for chromium in vegetables was radish leaf > coriander > radish root > dill. For lead, the order was dill = coriander = radish leaf > radish root and radish root had a boundary standard lead content for consumption (Table 5). Overall, radish root and dill had lower concentrations of heavy metals than radish leaf and coriander in Sanandaj. On the other hand, Finster et al. [55] have found that lead tends to concentrate more in radish roots rather than shoots in Chicago, USA. However, the same was not found in Sanandaj [55]. As shown in Table 5, coriander and dill absorb copper at higher rates than chromium, while for radish root and radish leaf it was reverse. All of the vegetables, except radish root, absorb lead at higher rates rather than cobalt. Meanwhile, all of the vegetables were poor absorber of arsenic and cadmium. It is noteworthy that Sipter et al. [56] have found that sorrel, carrot, and onion absorb cadmium and zinc at higher rates than lead. Although zinc was the most absorbed heavy metal after aluminum in Sanandaj’s sampled vegetables, lead and cobalt were absorbed at higher rates than cadmium. As illustrated in Figure 4, the vegetables of northeastern farms had more concentrations of aluminum, chromium, copper, and nickel while other measured heavy elements were lower in southwestern sites.

## Conclusions

In conclusion, despite the concentrations of measured heavy metals in soil and irrigation water were below the international standards, the concentrations of chromium and lead in vegetables were higher than FAO and the WHO standards.

## References

[1] Nagajyoti P, Lee K, Sreekanth T (2010). Heavy metals, occurrence and toxicity for plants: a review. Environ Chem Lett.

[2] Nriagu JO (1996). History of global metal pollution. Science.

[3] Järup L (2003). Hazards of heavy metal contamination. Br Med Bull.

[4] Tong S, von Schirnding YE, Prapamontol T (2000). Environmental lead exposure: a public health problem of global dimensions. Bull World Health Organ.

[5] Neff JM (1997). Ecotoxicology of arsenic in the marine environment. Environ Toxicol Chem.

[6] Duker AA, Carranza E, Hale M (2005). Arsenic geochemistry and health. Environ Int.

[7] Thompson J, Bannigan J (2008). Cadmium: toxic effects on the reproductive system and the embryo. Reprod Toxicol.

[8] Wagner GJ (1993). Accumulation of cadmium in crop plants and its consequences to human health. Adv Agron.

[9] Waisberg M, Joseph P, Hale B, Beyersmann D (2003). Molecular and cellular mechanisms of cadmium carcinogenesis. Toxicology.

[10] Denkhaus E, Salnikow K (2002). Nickel essentiality, toxicity, and carcinogenicity. Crit Rev Oncol Hematol.

[11] Zahir F, Rizwi SJ, Haq SK, Khan RH (2005). Low dose mercury toxicity and human health. Environ Toxicol Pharmacol.

[12] Nishijo M, Nakagawa H, Morikawa Y, Tabata M, Senma M, Miura K, Takahara H, Kawano S, Nishi M, Mizukoshi K (1995). Mortality of inhabitants in an area polluted by cadmium: 15 year follow up. Occup Environ Med.

[13] Hayashi C, Koizumi N, Nishio H, Koizumi N, Ikeda M (2012). Cadmium and other metal levels in autopsy samples from a cadmium-polluted area and Non-polluted control areas in Japan. Biol Trace Elem Res.

[14] Weiss B, Clarkson TW, Simon W (2002). Silent latency periods in methylmercury poisoning and in neurodegenerative disease. Environ Health Perspect.

[15] Staessen JA, Roels HA, Emelianov D, Kuznetsova T, Thijs L, Vangronsveld J, Fagard R (1999). Environmental exposure to cadmium, forearm bone density, and risk of fractures: prospective population study. Public health and environmental exposure to cadmium (PheeCad) study group. Lancet.

[16] Kolonel LN (1976). Association of cadmium with renal cancer. Cancer.

[17] Lanphear BP, Hornung R, Khoury J, Yolton K, Baghurst P, Bellinger DC, Canfield RL, Dietrich KN, Bornschein R, Greene T (2005). Low-level environmental lead exposure and children’s intellectual function: an international pooled analysis. Environ Health Perspect.

[18] Liu J-X, Zhou G-B, Chen S-J, Chen Z (2012). Arsenic compounds: revived ancient remedies in the fight against human malignancies. Curr Opin Chem Biol.

[19] Clemens S (2006). Toxic metal accumulation, responses to exposure and mechanisms of tolerance in plants. Biochimie.

[20] Volesky B, Holan Z (1995). Biosorption of heavy metals. Biotechnol Prog.

[21] Veglio F, Beolchini F (1997). Removal of metals by biosorption: a review. Hydrometallurgy.

[22] QuSheng L, ShaSha C, CeHui M, Bei C, LiHua P, FangBing Y (2010). Toxic effects of heavy metals and their accumulation in vegetables grown in a saline soil. Ecotoxicol Environ Saf.

[23] Clemens S, Palmgren MG, Krämer U (2002). A long way ahead: understanding and engineering plant metal accumulation. Trends Plant Sci.

[24] Maleki A, Zarasvand Alasvand M (2008). Heavy metals in selected edible vegetables and estimation of their daily intake in Sanandaj, Iran. Southeast Asian J Trop Med Public Health.

[25] Mohajer R, Salehi M, Mohammadi J (2012). Accumulation of cadmium and lead in soils and vegetables of Lenjanat Region in Isfahan Province, Iran. Int J Agronomy Plant Product.

[26] Eslami A, Khaniki GJ, Nurani M, Mehrasbi M, Peyda M, Azimi R (2007). Heavy metals in edible green vegetables grown along the sites of the Zanjanrood river in Zanjan, Iran. J Biol Sci.

[27] Pourang N, Noori AS (2012). Assessment of metals in fourteen species of vegetables and crops cultivated in a suburban area using multivariate analyses. Toxicol Environ Chem.

[28] Aghili F, Khoshgoftarmanesh A, Afyuni M, Schulin R (2009). Health risks of heavy metals through consumption of greenhouse vegetables grown in central Iran. Hum Ecol Risk Assess.

[29] Wang L, Guo Z, Xiao X, Chen T, Liao X, Song J, Wu B (2008). Heavy metal pollution of soils and vegetables in the midstream and downstream of the Xiangjiang River, Hunan Province. J Geogr Sci.

[30] Wei B, Yang L (2010). A review of heavy metal contaminations in urban soils, urban road dusts and agricultural soils from China. Microchem J.

[31] Akrong MO, Cobbina SJ, Ampofo JA (2012). Assessment of heavy metals in lettuce grown in soils irrigated with different water sources in the Accra metropolis. Res J Environ Earth Sci.

[32] Wang XL, Sato T, Xing BS, Tao S (2005). Health risks of heavy metals to the general public in Tianjin, China via consumption of vegetables and fish. Sci Total Environ.

[33] Bahemuka TE, Mubofu EB (1999). Heavy metals in edible green vegetables grown along the sites of the Sinza and Msimbazi rivers in Dar es Salaam, Tanzania. Food Chem.

[34] Karyab H, Mahvi AH, Nazmara S, Bahojb A (2013). Determination of water sources contamination to diazinon and malathion and spatial pollution patterns in Qazvin, Iran. Bull Environ Contam Toxicol.

[35] Amini H, Taghavi-Shahri SM, Henderson SB, Naddafi K, Nabizadeh R, Yunesian M (2014). Land use regression models to estimate the annual and seasonal spatial variability of sulfur dioxide and particulate matter in Tehran, Iran. Sci Total Environ.

[36] Hosseini G, Maleki A, Amini H, Mohammadi S, Hassanvand MS, Giahi O, Gharibi F (2014). Health impact assessment of particulate matter in Sanandaj, Kurdistan, Iran. J Adv Environ Health Res.

[37] Sridhara Chary N, Kamala C, Samuel Suman Raj D (2008). Assessing risk of heavy metals from consuming food grown on sewage irrigated soils and food chain transfer. Ecotoxicol Environ Saf.

[38] Weldegebriel Y, Chandravanshi BS, Wondimu T (2012). Concentration levels of metals in vegetables grown in soils irrigated with river water in Addis Ababa, Ethiopia. Ecotoxicol Environ Saf.

[39] Rice E, Baird R, Eaton A, Clesceri L (2012). Standard Methods for the Examination of Water and Waste Water.

[40] Tiwari K, Singh N, Patel M, Tiwari M, Rai U (2011). Metal contamination of soil and translocation in vegetables growing under industrial wastewater irrigated agricultural field of Vadodara, Gujarat, India. Ecotoxicol Environ Saf.

[41] El Bassam N, Tietjen C (1977). Municipal sludge as organic fertilizer with special reference to the heavy metals constituents. Soil Organic Matter Studies.

[42] McGrath S, Chang A, Page A, Witter E (1994). Land application of sewage sludge: scientific perspectives of heavy metal loading limits in Europe and the United States. Environ Rev.

[43] Maleki A, Gharibi F, Alimohammadi M, Daraei H, Zandsalimi Y (2014). Concentration levels of heavy metals in irrigation water and vegetables grown in peri-urban areas of Sanandaj, Iran. J Adv Environ Health Res.

[44] Codex Alimentarius Commission (FAO/WHO) (2001). Food Additives and Contaminants.

[45] Hernandez L, Probst A, Probst J, Ulrich E (2003). Heavy metal distribution in some French forest soils: evidence for atmospheric contamination. Sci Total Environ.

[46] US Environmental Protection Agency: **Ecological soil screening level for aluminum. Interim final.** OSWER Directive 9285. Washington, DC: 2003:7–60.

[47] Basta N, Ryan J, Chaney R (2005). Trace element chemistry in residual-treated soil. J Environ Qual.

[48] Mulder J, Rasmussen L, Driscoll C: **Aluminum chemistry of acidic sandy soils with various inputs of acidic deposition in The Netherlands and in Denmark.** In *Environmental Chemistry and Toxicology of Aluminum.* Edited by Lewis T. CRC Press; 1989:171–194.

[49] Finkelstein Y, Markowitz ME, Rosen JF (1998). Low-level lead-induced neurotoxicity in children: an update on central nervous system effects. Brain Res Rev.

[50] Givehchi R, Arhami M, Tajrishy M (2013). Contribution of the Middle Eastern dust source areas to PM10 Levels in urban receptors: case study of Tehran, Iran. Atmos Environ.

[51] Gharehchahi E, Mahvi AH, Amini H, Nabizadeh R, Akhlaghi AA, Shamsipour M, Yunesian M (2013). Health impact assessment of air pollution in Shiraz, Iran: a two-part study. J Environ Health Sci Eng.

[52] Singh HP, Mahajan P, Kaur S, Batish DR, Kohli RK: **Chromium toxicity and tolerance in plants.** *Environ Chem Lett* 2013, 1–26.

[53] Shanker AK, Cervantes C, Loza-Tavera H, Avudainayagam S (2005). Chromium toxicity in plants. Environ Int.

[54] Costa M, Klein CB (2006). Toxicity and carcinogenicity of chromium compounds in humans. CRC Crit Rev Toxicol.

[55] Finster ME, Gray KA, Binns HJ (2004). Lead levels of edibles grown in contaminated residential soils: a field survey. Sci Total Environ.

[56] Sipter E, Rózsa E, Gruiz K, Tátrai E, Morvai V (2008). Site-specific risk assessment in contaminated vegetable gardens. Chemosphere.

